# Time-Dependent Ultrafast Quadratic Nonlinearity in an Epsilon-Near-Zero Platform

**DOI:** 10.1021/acs.nanolett.4c00282

**Published:** 2024-03-14

**Authors:** Anton Yu. Bykov, Junhong Deng, Guixin Li, Anatoly V. Zayats

**Affiliations:** † Department of Physics and London Centre for Nanotechnology, King’s College London, London WS2R 2LS, U.K.; ‡ Shenzhen Institute for Quantum Science and Engineering, Southern University of Science and Technology, Shenzhen 518055, China; ¶ Department of Materials Science and Engineering, Southern University of Science and Technology, Shenzhen 518055, China

**Keywords:** hot-carrier dynamics, epsilon-near-zero materials, plasmonic metasurfaces, second harmonic generation, ultrafast all-optical modulation

## Abstract

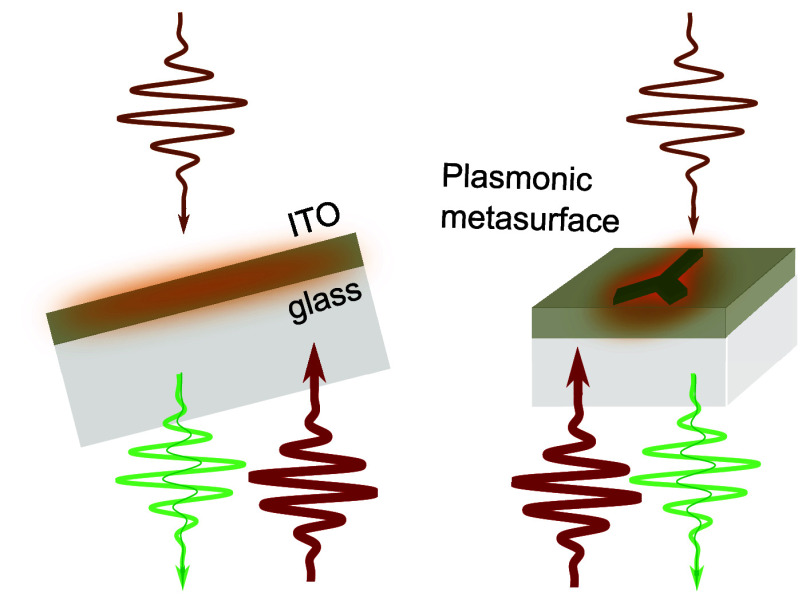

Ultrafast nonlinearity, which results in modulation of the linear optical response, is a basis for the development of time-varying media, in particular those operating in the epsilon-near-zero (ENZ) regime. Here, we demonstrate that the intraband excitation of hot electrons in the ENZ film results in a second-harmonic resonance shift of ∼10 THz (40 nm) and second-harmonic generation (SHG) intensity changes of >100% with only minor (<1%) changes in linear transmission. The modulation is 10-fold enhanced by a plasmonic metasurface coupled to a film, allowing for ultrafast modulation of circularly polarized SHG. The effect is described by the plasma frequency renormalization in the ENZ material and the modification of the electron damping, with a possible influence of the hot-electron dynamics on the quadratic susceptibility. The results elucidate the nature of the second-order nonlinearity in ENZ materials and pave the way to the rational engineering of active nonlinear metamaterials and metasurfaces for time-varying applications.

Nonlinear optical processes lie at the center of many optical technologies and enable a range of practical applications such as quantum technologies,^1^ telecommunication, laser processing, optical switching,^2,3^ etc. Very recently, they have also been used to design time-varying photonic media, where the strong modulation of a refractive index takes place at the time scales of the wave period or pulse coherence time, allowing temporal control of electromagnetic wave propagation.^4^ In all the cases, the applications are fundamentally limited by the existing materials universally exhibiting weak nonlinearities, especially if the ultrafast response is required. Various approaches for enhancing nonlinear interactions by nanostructuring have been exploited, such as using artificial materials that lack inversion symmetry^5,6^ or using the local field enhancement in the vicinity of plasmonic^7−9^ or Mie^10^ resonances.

Natural and metastructured epsilon-near-zero (ENZ) media have recently attracted attention as a means of enhancing light–matter interaction on the nanoscale and subsequently boosting parametric (coherent) nonlinear optical effects^11−15^ and realizing strong power-dependent modulation of the refractive index.^16−20^ To date, ultrafast control over coherent optical nonlinearities in ENZ materials and/or nanostructures has not been demonstrated.

In the near-infrared spectral range, indium tin oxide (ITO) has emerged as an ENZ material of choice that can sustain a high pump fluence without damage and provide strong modulation of the refractive index with very short subpicosecond lifetimes,^21,22^ enabling the development of novel time-varying materials and structures. Multiple novel nonlinear optical phenomena within this paradigm have been demonstrated in ITO, including time refraction,^23,24^ nonperturbative four-wave mixing,^25^ and temporal analogy of optical diffraction.^26,27^ The debate over the microscopic mechanisms of second- and third-order nonlinearities in ITO is ongoing with different approaches applying either constant, wavelength-independent bulk nonlinearities^11,14^ or surface free-electron nonlinearities, treated semiclassicaly within the scope of the hydrodynamic model with the inclusion of hot-electron effects.^13^

Here, we demonstrate the strong modulation of the quadratic nonlinear response of a deeply subwavelength (10 nm thick) film of ITO near the ENZ condition, related to the excitation of hot electrons. Compared to that of a bare ITO film, the modulation is 10-fold enhanced in the plasmonic ITO metasurface with >100-fold enhanced overall SHG, due to the fundamental field reshaping. By examining the polarization properties of the SHG and the all-optical modulation behavior, we demonstrate the role of a free-electron nature of ITO quadratic nonlinearity and the importance of both the plasma frequency renormalization and the electron–electron scattering modification (enhanced compared to conventional metals), with a potential small effect of the dependence of the second-order susceptibility on hot-electron excitation. The results show that the coupled second- and third-order free-electron nonlinearities can be efficiently used to modulate parametric nonlinear optical processes in ITO in the ENZ spectral range.

The ITO nanofilms and plasmonic ITO metasurfaces were fabricated as described in detail in ref (14). The optical constants of the ITO nanolayer have been measured via spectroscopic ellipsometry (see section 1.1 of the Supporting Information) and fitted with the Drude–Lorentz dispersion model, identifying the values of the bulk plasma frequency (ω_p_ = 2.17 eV) and bulk damping parameter in the infrared (γ = 0.07 eV). The ITO nanofilm exhibits ENZ behavior with a refractive index *n* = 0.49 + 0.45i at a wavelength of ∼1160 nm.

Static nonlinear optical characterization of the nanostructure was performed using a tunable (1050–1500 nm) output of the optical parametric amplifier (OPA, Light Conversion Orpheus HP, 150 fs pulses), which was expanded in a dispersionless mirror-based 4× beam expander and focused on the ITO film from air with the help of a 50 mm focal length lens, producing a spot with a diameter of ∼15 μm. The nonlinear optical response from a bare ITO nanofilm was analyzed at an angle of incidence of 30° with a linearly polarized fundamental and SHG light. For the nonlinear metasurface, the experiments were performed at normal incidence, in the circular polarization basis, similar to the geometry previously used for the studies of the enhanced static SHG response.^14^ The generated SH light transmitted through the glass substrate, following the polarization analysis, was routed into the IsoPlane spectrometer equipped with the cooled PIXIS256 CCD camera, which was also simultaneously monitoring the SHG signal generated in the reference arm from the surface of the z-cut quartz crystal to monitor potential changes in the nonlinear signal caused by the changes in the excitation light power, pulse duration, or angular width.^28^

Transient absorption and transient SHG measurements were performed in a collinear geometry by adding an infrared control beam (1028 nm wavelength) for the excitation of hot carriers in ITO. The output of the Yb:KGW amplifier (Light Conversion Pharos, 250 fs pulses at 600 kHz) was focused with a 35 mm focal length lens through the substrate, forming a spot with a diameter of ∼20 μm. In addition to the SHG, the transmitted fundamental (acting as a probe in the transient SHG measurements) light was recorded with the help of a biased InGaAs photodiode and a lock-in amplifier locked to the frequency of the optical chopper placed in the control beam, providing a linear transient absorption signal. A second biased photodiode was used to monitor the variation of the probe power for transient absorption spectroscopy (see section 1.2 of the Supporting Information for details).

Figure 1a shows the static SHG spectrum of the ITO nanofilm in p-in/p-out and s-in/p-out polarization configurations. Like the previously reported results, the SHG exhibits strong, 20-fold enhancement near the ENZ wavelength, compared to nonresonant excitation due to the enhanced discontinuity of the normal component of the fundamental field at the interface between air and the ENZ medium. A much weaker second harmonic signal is detected in the s-in/p-out polarization combination, demonstrating the negligible contribution of χ_*zxx*_^(2)^ = χ_*zyy*_^(2)^ components of quadratic nonlinear optical susceptibility (see section 3.1 of the Supporting Information for details). Comparison of the polar plots of the SHG intensity, measured with crossed and aligned fundamental and SHG polarizations, shows, however, a clear presence of non-zero χ_*xzx*_^(2)^ = χ_*yzy*_^(2)^ components of the second-order susceptibility of ITO (Figure 1b). These components are often overlooked despite being previously reported.^11^

**Figure 1:**
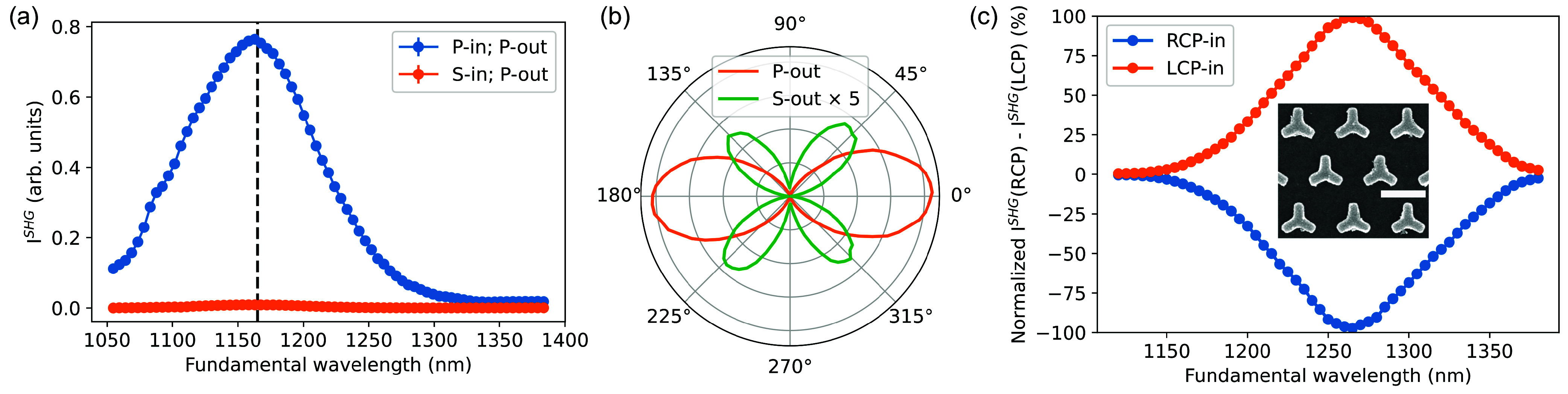
SHG spectroscopy. (a) SHG spectra from the ITO nanofilm in the vicinity of the ENZ wavelength measured at an angle of incidence of 30° for different polarization configurations. The dashed vertical line indicates the ENZ wavelength determined from spectroscopic ellipsometry. (b) Polar plots of the SHG intensity obtained by rotating the polarization of fundamental light while keeping the SHG polarization fixed. (c) Circular polarization-selective resonant SHG spectra from the plasmonic ITO metasurface consisting of 3-fold-symmetry plasmonic nanoantennas measured at normal incidence. The inset shows a scanning electron microscopy image of the metasurface (scale bar of 500 nm).

The nonlinear optical response of the plasmonic ITO metasurface, investigated in the circular polarization basis, shows an ∼100-fold enhanced SHG at the resonant wavelength compared to the nonresonant excitation, in contrast to a 20-fold enhancement in the bare ITO nanofilm, because of the reshaping of the polarization of the fundamental field (Figure 1c), in agreement with previous reports.^14^ The SHG peak is red-shifted due to coupling of the ENZ resonance of ITO with the plasmonic mode of the nanoantennas and has almost unitary circular selectivity determined by the SHG selection rules for a medium possessing 3-fold rotational symmetry (see eq S4 of the Supporting Information).

To achieve transient SHG (Figure 2), the nanofilm was modulated using a photon energy of 1.2 eV (1028 nm wavelength), which cannot excite electron–hole pairs in ITO due to the interband optical transitions even with the three-photon absorption, because the optical band gap of ITO is ∼3.75 eV.^29,30^ However, hot electrons can be excited upon intraband absorption. Under moderate photoexcitation conditions (fluence of 5 mJ/cm^2^), an almost 40 nm (∼10 THz) shift and broadening of the resonant SHG peak were observed with the relative SHG intensity changes at a slope exceeding 100%, while only minor, less than 1% changes are observed in the linear response. Suppression of the absolute SHG signal is also evident.

**Figure 2:**
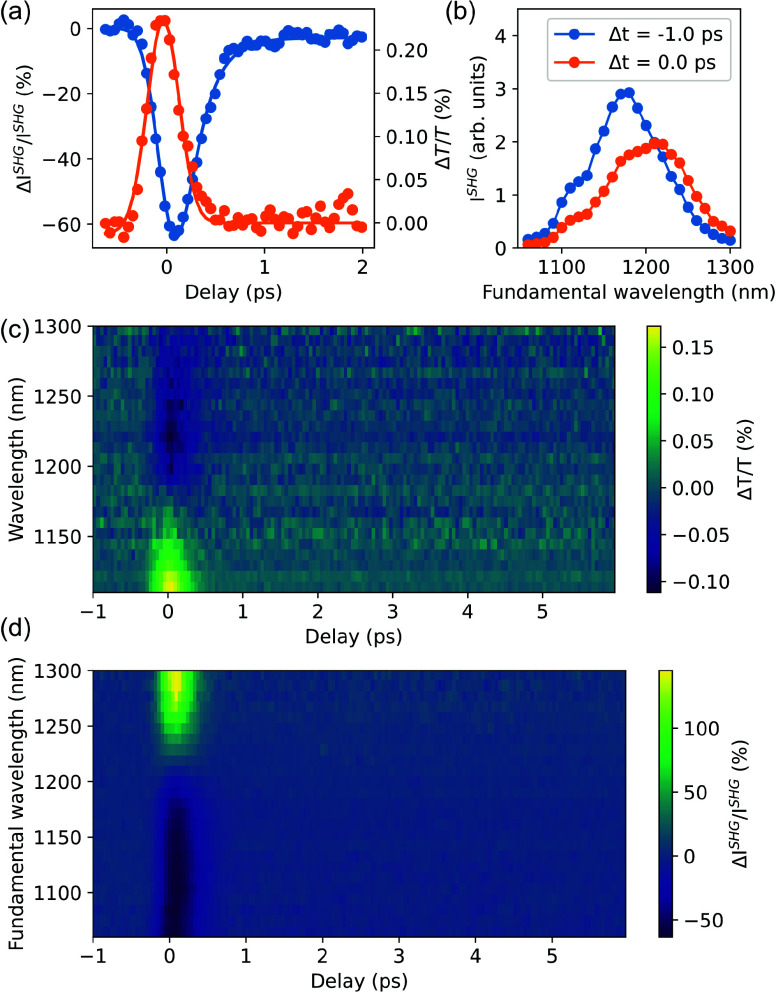
Ultrafast control of quadratic nonlinearity in the ITO nanofilm. (a) Linear (orange) and second-harmonic (blue) pump–probe traces at a fixed wavelength of 1100 nm under the identical excitation conditions. (b) Shift of the ENZ SHG resonance under hot-electron excitation. (c) Transient absorption and (d) transient SHG spectra.

The induced modulation is short-lived, decaying on a time scale of <1 ps, when the hot-electron population completely dissipates the energy due to the heat exchange with the lattice (Figure 2a). Such fast cooling of a hot-electron gas in ITO is consistent with the previous observations^16^ and can be explained considering both the low free-carrier density, which results in a smaller free-carrier specific heat, and the high Debye frequency, which is approximately an order of magnitude higher than in conventional noble metals.^30^ Deconvolution of the linear pump–probe traces obtained at increasing excitation powers shows the power dependence of the decay time consistent with the predictions of the two-temperature model (TTM) and allows identification of an internal relaxation time on the order of 150 fs (see section 2 of the Supporting Information). We also emphasize that while the contribution of degenerate four-wave mixing may influence the transient linear absorption, the SHG modulation would require forth-order nonlinearity, which is symmetry-forbidden in the bulk of ITO, and therefore, the strong modulation observed here cannot be explained by parametric nonlinearities.

Theoretical analysis of the transient linear and nonlinear response of ITO was performed using the TTM, which describes the temporal response of the photoexcited hot-electron population in ITO (see section 4 of the Supporting Information). The hot-electron excitation results in the renormalization of the plasma frequency due to the change in the effective mass of the hot electrons in the nonparabolic conduction band of ITO.^15,18,22^ This effect produces an effective hot-electron temperature (*T*_e_) dependent plasma frequency that modifies the optical response:^22^
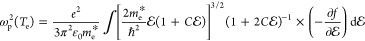
1where *f* is the Fermi function, *C* = 0.42 eV^–1^ is the nonparabolicity factor of the conduction band of ITO, and *m*_e_^*^ = 0.4*m*_*e*_ is the effective electron mass at the bottom of the conduction band (*m*_e_ is the free-electron mass).^18^ It is important to note that this mechanism of the hot-electron-dependent optical response is different from that of the hot-electron-induced change in the Drude damping of conventional metals, which is traditionally explained by electron temperature-dependent fractional Umklapp electron–electron scattering.^31^

In ITO, the main contribution to the Drude damping comes from scattering on charged impurities and grain boundaries,^22,32^ with minor corrections imposed by the conventional electron–phonon and Umklapp electron–electron scattering, which are commonly disregarded for steady-state measurements. However, at increased electron temperatures, the  dependence of the electron–electron scattering makes it essential to explicitly consider the transient change of the Drude damping constant (see section 4.1 of the Supporting Information for derivations):
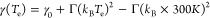
2where *k*_B_ is the Boltzmann constant, Γ = 0.48 eV^–2^ fs^–1^ and γ_0_ is the electron temperature-independent scattering constant that contains the contributions of electron–phonon and electron–impurity scattering and the temperature-independent part of electron–electron scattering (see section 4.1 of the Supporting Information). While electron–phonon scattering depends, in principle, on the temperature of the lattice, we neglect this effect because in our experiments almost no transient signals were observed after 1 ps (Figure 2a–d), suggesting that the increase in the lattice temperature does not contribute substantially to the measured optical constants.

These effects upon the excitation of hot electrons result in a change in the refractive index of ITO (Δ*n* ≈ 0.16–0.07i) at the static ENZ wavelength of 1160 nm and a shift of the ENZ wavelength to 1200 nm (Figure 3a, inset) at which *n* = 0.5 + 0.5i is reached (Δ*n* ≈ 0.14–0.12i).

The model that takes into account the renormalization of the plasma frequency as the main mechanism and the modification of the Drude damping constant under hot-carrier excitation explains well the behavior of the observed ultrafast modulation of both linear and nonlinear optical signals (Figure 3). The calculated hot-carrier temperature dependence shows that under the experimental photoexcitation conditions the temperature of hot carriers reaches 2800 K (Figure 3a). This, in turn, red-shifts the plasma frequency of the ITO by ∼60 meV, producing a weak modulation of the linear optical transmission of a thin ITO film, spectrally consistent with the experimental data [cf. Figure 3c (orange) and Figure 2c]. The simulated linear transient behavior shows a good agreement with the experimental measurements without any free parameters.

**Figure 3:**
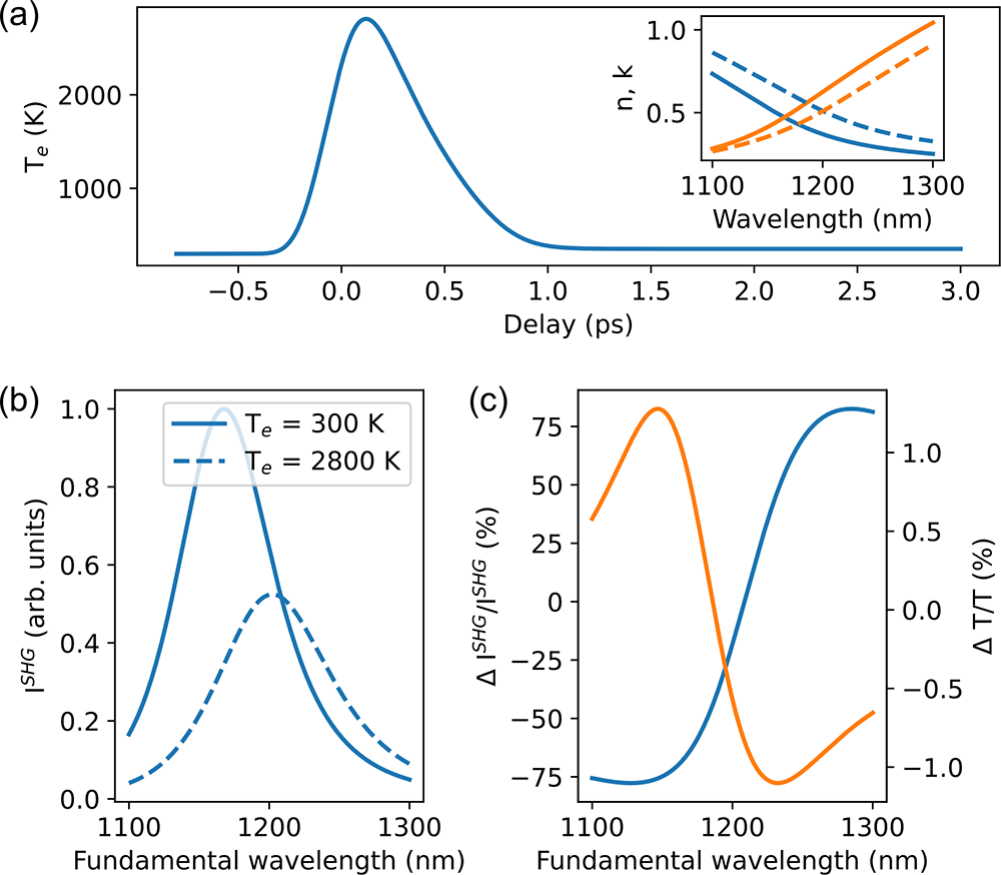
Modeling ultrafast nonlinearity in ITO. (a) Time dependence of the hot-electron temperature simulated using the TTM under the experimental conditions. The inset shows the real (blue) and imaginary (orange) parts of the complex refractive index of ITO in the ground (solid) and excited (dashed) states. (b) Simulated shift of the ENZ SHG resonance under hot-electron excitation. (c) Simulated spectra of SHG intensity modulation (blue) and linear transmission modulation (orange) under hot-electron excitation.

Similarly, the nonlinear simulations (see section 3.2 of the Supporting Information for details) reveal the SHG enhancement at the ENZ wavelength and the SHG peak red-shift from the renormalization of the plasma frequency inferred from modeling of the hot-electron dynamics, qualitatively reproducing the experimental data (cf. Figure 3b and Figure 2b). We therefore conclude that a relatively weak optical excitation of hot electrons in the ITO nanofilm is indeed manifested in a dramatic modification of the ENZ SHG.

An intriguing possibility is to consider the direct influence of the hot-electron dynamics on the nonlinear susceptibilities, enabled in the ENZ regime. Indeed, within the free-electron model, the surface nonlinear dipoles are^33−36^
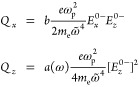
3where a common parametrization *a*(ω) and *b* by Rudnick and Stern is used,^33^*E*_*x*_^0–^ and *E*_*z*_^0–^ are the fundamental field components evaluated just inside the material, and . With both ω_p_ and γ being dependent on the electron temperature (eqs 1 and 2), we should anticipate modifications of the surface nonlinearities of ITO due to hot-electron excitation. The change in ω_p_ is estimated to account for an ∼5% modulation of the SHG intensity under our experimental conditions.

Finally, we demonstrate the ultrafast modulation of an ENZ-enhanced circularly selective SHG in the plasmonic ITO metasurface at normal incidence (Figure 4). The 10-fold enhanced modulation efficiencies, compared to the modulation that can be achieved with a bare nanofilm at the same control fluence, are observed in the same geometry as described above, except for normal incidence and the circular polarization basis for the fundamental and SHG waves. This enhancement is attributed to the combined action of the increased absorption of an ITO layer due to coupling to the optical resonances of the nanoantenna and the increased sensitivity of the optical and nonlinear optical response of the metasurface to the modulation of the optical constants of ITO due to the efficient conversion of the energy of the incoming fundamental light into plasmonic near fields, which is the same mechanism that is responsible for the strong enhancement of the static SHG response of the metasurface at normal incidence.

**Figure 4:**
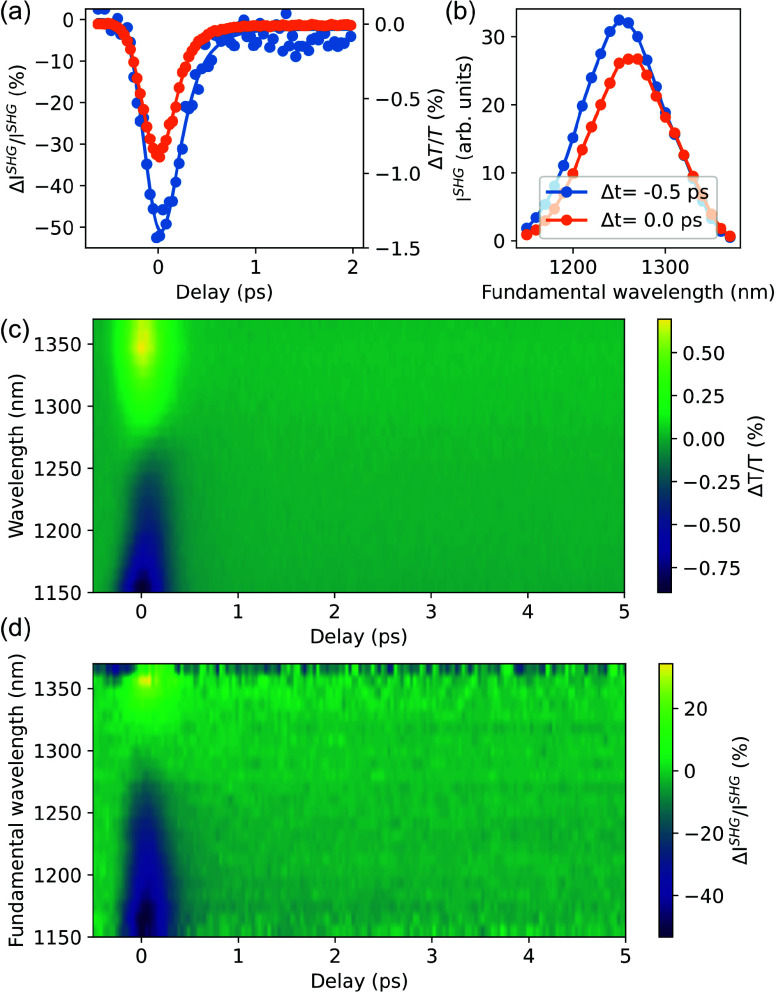
Ultrafast control of nonlinearity in the plasmonic ITO metasurface. (a) Temporal dependence of linear transmission (orange) and SHG (blue) at a fixed wavelength of 1150 nm. (b) Shift of the ENZ SHG resonance under hot-electron excitation. (c) Transient absorption and (d) transient SHG spectra. The illumination conditions are similar to those depicted in Figure 2 but with an order of magnitude weaker control fluence of 0.4 mJ/cm^2^.

While the gold nanostructures deposited on ITO did enhance the static and dynamic nonlinear responses of the ITO film, no difference is observed in the temporal behavior of the linear transmission and the SHG. This, in agreement with the theoretical calculations, confirms that most of the energy of the control beam is absorbed in the ITO and the dynamics of the hot electrons in gold does not contribute to the observed effects. Therefore, the same mechanism that is responsible for the SHG modulation in an ITO nanofilm leads to the enhanced modulation of the SHG from the metasurface. The SHG polarization analysis also did not detect the polarization changes that one would expect if the linearly polarized control light breaks the 3-fold symmetry due to hot-electron excitation in Au nanostructures. It should be noted that the modulation of the SHG from purely plasmonic metasurfaces was previously observed under stronger excitation conditions, resulting in addition in four-wave mixing between control and fundamental light.^37^ The latter is not observed in our experiments with ITO as the fundamental light intensity is 5 times weaker than the control light intensity. The plasmonic ITO metasurface with the nanostructures of 3-fold rotational symmetry brings about a pronounced circular selectivity for the quadratic nonlinear optical response (Figure 1c), and the modulation scheme demonstrated here provides an opportunity for the control of circularly polarized SHG in a metasurface platform.

In conclusion, we demonstrate all-optical control of the quadratic optical nonlinearity of an ENZ material due to hot-electron excitation. A substantial shift and broadening of the ENZ second-harmonic resonance under moderate photoexcitation conditions were observed, controlled by the hot-electron contribution to the plasma frequency renormalization and the Drude damping modification due to the stronger effect of fractional Umklapp electron–electron scattering in ITO. These processes result in ultrafast modulation of the permittivity with potentially a small contribution from the second-order susceptibility modification by hot-electron excitation. A microscopic model of the hot-electron nonlinear dynamics in ITO, taking into account the nonparabolic conduction band of ITO and hot-electron temperature-dependent chemical potential, electron–electron scattering, was applied for the first time to describe transient second-harmonic generation, showing a good agreement with the experimental behavior without free parameters. The results provide insight into the fundamentals of enhanced quadratic nonlinearities in transparent conductive oxides, are instrumental in the design of active nonlinear metasurfaces based on ENZ materials, and pave the way toward realization of nonlinear time-varying media.

## Data Availability

All of the data supporting the findings of this study are presented in the Results section and the Supporting Information and available from the corresponding authors upon reasonable request.

## Supporting Information Available

Experimental characterization of the ITO nanofilms, description of the pump–probe spectroscopy setup, analysis of the intrinsic hot-carrier relaxation time in ITO, and modeling of hot-carrier dynamics and the quadratic nonlinear optical response (PDF)
