# Supplementary material for: Time-Dependent Ultrafast Quadratic Nonlinearity in an Epsilon-Near-Zero Platform
Source: Nano Lett. 2024 Mar 14;24(12):3744–9. doi: 10.1021/acs.nanolett.4c00282 (PMC10979426; doi:10.1021/acs.nanolett.4c00282)
Supplement: Supplementary file 1 — nl4c00282_si_001.pdf [file nl4c00282_si_001.pdf]

Supporting Information
Time-dependent ultrafast quadratic nonlinearity in an epsilon-near-zero platform

Anton Yu. Bykov,[*,†] Junhong Deng,[‡] Guixin Li,[¶] and Anatoly V. Zayats[†]

†*Department of Physics and London Centre for Nanotechnology, King's College London, London, WS2R 2LS, UK*

‡*Shenzhen Institute for Quantum Science and Engineering, Southern University of Science and Technology, Shenzhen 518055, China*

¶*Department of Materials Science and Engineering, Southern University of Science and Technology, Shenzhen 518055, China*

E-mail: anton.bykov@kcl.ac.uk

1. Experimental methods

1.1 ITO ellipsometry

To ensure accurate determination of the dielectric constants, spectroscopic ellipsometry of the ITO nanofilm was carried out. Initially, the glass substrate was characterized separately and the determined optical constants were found to be close to the tabulated values for BK7 glass (Fig. S1(a)). Subsequently, the ellipsometric spectra of the ITO film on a substrate were fitted to determine the dielectric constants and the film thickness using the Durde-Lorentz model (Fig. S1(b)). An ITO film thickness of 11 nm and the parameters of the Drude model, described in the main text, were obtained (Fig. S1(c)). The value of $\epsilon_\infty \approx 4.1$,

which takes into account the tails of the Lorenz oscillators, was obtained from the fit in line with the previous reports on ITO,[1,2] and a Drude damping of 0.07 eV also quantitatively match the other studies.[3] The surface roughness of the order of 0.5 nm, obtained from the results of scanning atomic force microscopy (AFM) was included in the modelling and did not improve the fitting of ellisometric data. We therefore ignored it in the following calculations.

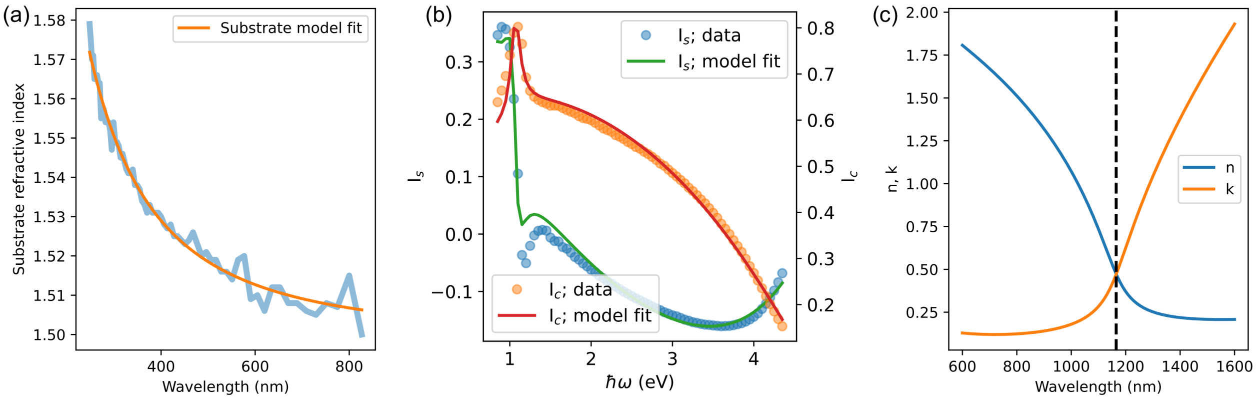

Figure S1: (a) Experimental refractive index of the substrate and the model fit. (b) Measured ellipsometric parameters $I_s = \sin 2\Psi \sin\Delta$ and $I_c = \sin 2\Psi \cos\Delta$, and the respective Drude-Lorenz model fits. (c) Complex refractive index of ITO, obtained from (b), showing an epsilon-near-zero condition around a 1160 nm wavelength.

1.2 Pump-probe spectroscopy

The transient optical measurements were performed in a collinear configuration with a 1028 nm output of an Yb:KGW amplifier (Light Conversion Pharos, 250 fs pulses at 600 kHz) as a control beam and a tuneable output of an OPA (Light Conversion Orpheus, 150 fs) as a probe beam. The path length difference between the pump and probe beams is adjusted using a mechanical delay line. The probe beam is divided into a fundamental (used for SHG excitation and two reference beams. The fundamental and control beams were made collinear and counter-propagating by a mirror arrangement and focused on the sample: the fundamental beam from the air/ITO interface and the control beam from the glass/ITO interface, into spots around 15 and 20 μm diameters, respectively. Transmitted through the sample fundamental beam and one of the two reference beams are used to measure a

transient linear absorption with two InGaAs photodiodes, monitored with a lock-in amplifier (Stanford Research SR865A), which uses as its reference the frequency of an optical chopper positioned in the pump path. The second reference beam was used to generate the second-harmonic signal from the surface of a thick z-cut quartz crystal that is known to have no spectral features in the studied wavelength range. The reference SHG signal and the SHG signal generated from the sample were made parallel by the mirror arrangement and routed to the Princeton Instruments IsoPlane spectrometer equipped with the cooled PIXIS256 CCD camera for simultaneous detection. The reference SHG beam was used to monitor potential changes in a nonlinear signal caused by the changes in the excitation light power, pulse duration or angular width.[4]

The probe wavelength was tuned in the range 1050–1500 nm, which corresponds to the spectral range of epsilon-near-zero regime of ITO and the metasurfaces studied here. The schematics of the optical setup is shown in Fig. S2. It allows simultaneous measurements of linear and SHG transient responses in transmission.

2. Hot-carrier relaxation time in ITO

Figure S3(a,b) shows the power-dependent transient transmission of a plasmonic-ITO metasurface measured at normal incidence. A clear increase in the hot-electron decay time is observed with the increase of the pump power. Deconvolution of the traces with the Gaussian instrument response function, corresponding to the cross-correlation trace of the pump and probe pulses, allows to determine a weak excitation limit of the hot-electron lifetime in the ITO film of the order of 150 fs (Fig. S3(b)).

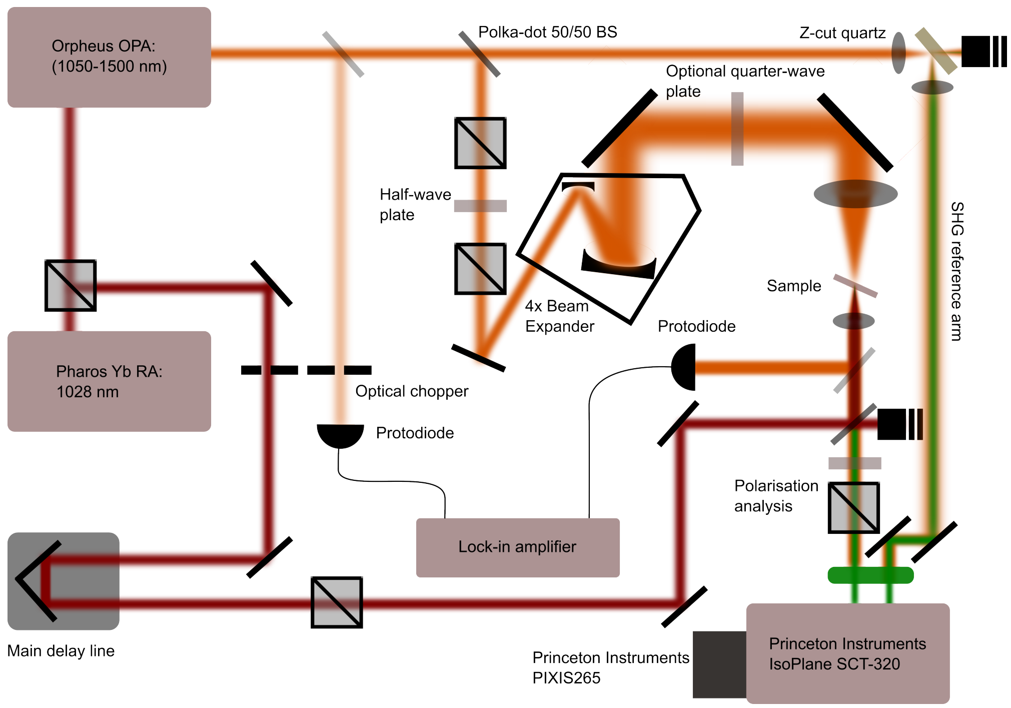

Figure S2: Schematics of the experimental setup for simultaneous measurements of transient linear absorption and transient SHG.

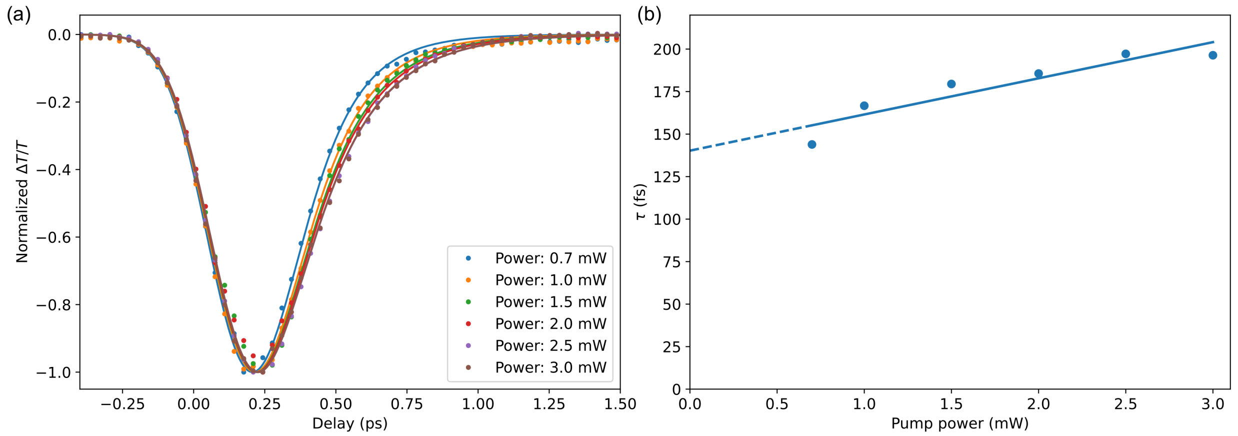

Figure S3: (a) Normalized pump-probe traces measured at normal incidence from the plasmonic-ITO metasurface at different pump powers (control wavelength 1028 nm, probe wavelength 1150 nm). (b) Power dependence of the deconvoluted decay times in ITO. Line is the best fit to the experimntal points.

3. Modelling quadratic nonlinear-optical response

3.1 Polarisation analysis of static SHG

Second-harmonic generation from metal surfaces has been extensively studied using phenomenological,[5,6] hydrodynamic[7,8] and quantum-mechanical[9] approaches. Recently, a hydrodynamic description has also been developed for ITO which suggests that symmetry considerations similar to those for free-electron metals may be responsible for the nonlinear response.[10] In metals, a phenomenological surface quadratic response is described by

$$\mathbf{P}^{2\omega} = \epsilon_0 \left[(\delta - \beta - 2\gamma)(\mathbf{E}\nabla)\mathbf{E} + \beta\mathbf{E}(\nabla\mathbf{E}) + \gamma\nabla(\mathbf{E}\cdot\mathbf{E}) \right], \tag{S1}$$

where ϵ_0 is the vacuum permittivity, $\mathbf{E}$ is the electric field of the fundamental (frequency ω) driving wave, and δ, β, and γ are the dimensionless constants. The first two terms in Eq. (S1) represent surface nonlinearities, related to the discontinuity of the normal component of the driving electric field, and can be related to $\chi^{(2)}_{zzz}$ and $\chi^{(2)}_{xzx} = \chi^{(2)}_{yzy}$ second-order nonlinear susceptibility components from the microscopic theory.[6] The last term is the bulk quadrupolar contribution that is present in the bulk of the media due to the permittivity dispersion. However, it is clear that this term produces p-polarised SHG for s-polarised fundamental light and could be, therefore, neglected in our case as discussed in the main text.

The polarisation analysis of the SHG from ITO was performed by varying the angle of polarisation of the incident fundamental wave $\mathbf{E}_{in} = \mathbf{p}E\sin(\theta) + \mathbf{s}E\cos(\theta)$ for a fixed s- or p-polarisation of the SH wave. Ignoring for simplicity the interface transmission/reflection coefficients, the polarisation dependence of the SH intensity can be obtained as

$$\begin{aligned} I_p^{2\omega} &\propto \left| \frac{k_x}{k^2 K}(k_z K_z \chi^{(2)}_{xzx} + k_x K_x \chi^{(2)}_{zzz}) \cos^2\theta \right|^2 \\ I_s^{2\omega} &\propto \left| \chi^{(2)}_{xzx} \frac{k_x}{k} \sin 2\theta \right|^2 \end{aligned} \tag{S2}$$

with k and K being the wave vectors of fundamental and SH waves, respectively. Equations (S2) correctly reproduce the experimental 2-fold and 4-fold dependences (Fig. 1(b)) confirming that both $\chi^{(2)}_{zzz}$ and $\chi^{(2)}_{xzx} = \chi^{(2)}_{yzy}$ components of the susceptibility are present in the nonlinear response of ITO (similar to the components arising at metal surfaces due to the free-electron nonlinearity).

For the metasurfaces with 3-fold rotation symmetry, the selectivity to the circularly-polarised illumination stems directly from the rotational symmetry of the nanostructures (Fig. 1(c)). The non-zero components of the quadratic susceptibility tensor for the effective medium possessing 3-fold rotational symmetry on the surface (which corresponds to the pyramidal C_{3v}(3m) point group) are[11]

$$xzx = yzy; xxz = yyz; zxx = zyy; zzz; yyy = -yyx = -xxy = -xyx \,. \tag{S3}$$

Applying the same procedure as above, the following expressions for the amplitudes of the SH electric field at normal incidence can be obtained (in the Jones matrix representation, ignoring E_z):

$$\begin{aligned} \mathbf{E}^{2\omega}_{RCP-in} &\propto \hat{\chi}^{(2)} : \begin{pmatrix} 1 \\ i \end{pmatrix}^2 = 2i\chi^{(2)}_{yyy} \begin{pmatrix} 1 \\ -i \end{pmatrix} \\ \mathbf{E}^{2\omega}_{LCP-in} &\propto \hat{\chi}^{(2)} : \begin{pmatrix} 1 \\ -i \end{pmatrix}^2 = 2i\chi^{(2)}_{yyy} \begin{pmatrix} 1 \\ i \end{pmatrix} \,. \end{aligned} \tag{S4}$$

The expressions in Eq. (S4) clearly demonstrate that the circularly-polarised fundamental fields will only produce the SH waves of exactly opposite helicity.

3.2 Modelling SHG from an ITO nanofilm

Nonlinear-optical response of a bare ITO nanofilm was modelled using a modified transfer-matrix method (TMM).[12] The electric field amplitude of the fundamental wave inside the ITO layer on a glass substrate is given by

$$\begin{aligned}\mathbf{E}(z) =& t_{12}m(\mathbf{u}_{2+}e^{ik_{z,2}z} + r_{23}e^{2ik_{z,2}d}\mathbf{u}_{2-}e^{-ik_{z,2}z}) \\ m =& (1 + r_{23}r_{12}e^{2ik_{z,2}d})^{-1},\end{aligned} \tag{S5}$$

where indices 1, 2, and 3 refer to air, ITO and a glass substrate, respectively, d is the thickness of the ITO layer, $k_{z,2}$ is the z-component of the wave vector in ITO, $\mathbf{u}_{2\pm} = \mathbf{p}_{2\pm}$ or $\mathbf{s}_{\pm}$ denotes the polarisation vectors for forward (+) and backward (-) propagating waves, and r_{ij} and t_{ij} represent the Fresnel reflection and transmission amplitudes, respectively, at the interfaces between media i and j. After the fundamental fields are evaluated inside the ITO film, they are combined with the nonlinear susceptibilities, as discussed in Section 3.1, to obtain the sources of the nonlinear polarisation.

Given the centrosymmetric crystal structure of ITO and the relevant nonlinear susceptibility components, the SHG response of an ITO film arises due to glass/ITO and ITO/air interfaces. To treat them analytically, one may position the nonlinear dipole sheets just outside the ITO layer. The calculation of the SHG field in the far field may then proceed through the standard approach of optics of stratified media:[12]

$$E_{3+} = s_+^f T_{12}T_{23}e^{iK_{z,2}d}M + s_+^b + s_-^b(R_{31} + T_{31}R_{21}T_{23}e^{iK_{z,2}d}M)\,, \tag{S6}$$

where R, T, M and K_z are defined the same ways as their lowercase counterparts above but at the second-harmonic frequency and

$$s_{\pm} = \frac{2\pi i k_0^2}{\kappa}\mathbf{u}_{\pm}\mathbf{P}^{NL} \tag{S7}$$

corresponds to the source terms defined on the front (air/ITO) and back (ITO/glass) interfaces. The potential impact of surface roughness on lowering surface quadratic nonlinearity[5] is implicitly included in the model within the best-fit values for the phenomenological $\chi^{(2)}$ nonlinear susceptibilities.

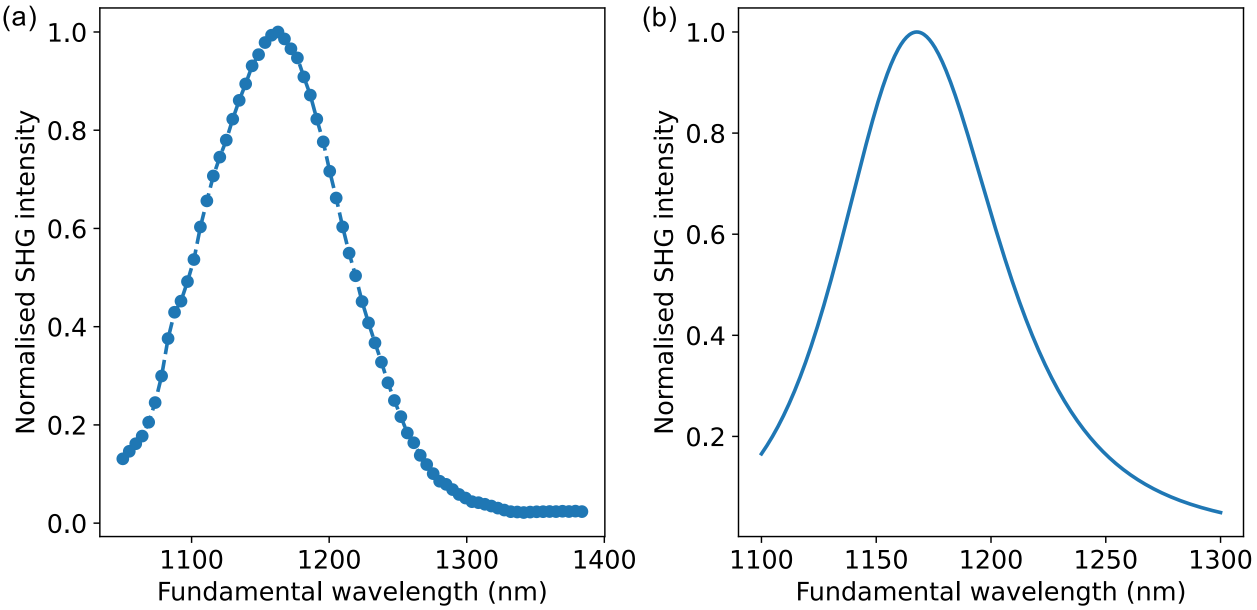

Figure S4: (a) Experimental and (b) calculated SHG spectra from the ITO film in p-in/p-out polarisation configuration at 30° angle of incidence. The ratio between the nonlinear susceptibilities was adjusted to obtain the best fit for the position of the experimental SHG peak: $\chi^{(2)}_{zzz}/\chi^{(2)}_{xzx}$ = -7.

4. Modelling hot-carrier dynamics

The effect of the control light illumination at a wavelength of 1028 nm was simulated with the TMM. Taking into account refraction in a glass substrate, a power absorption coefficient in the ITO film is approximately 2.6%. The hot-electron dynamics after absorption was modelled with the two-temperature model (TTM) in the usual way:[13]

$$\begin{aligned} C_e\left(T_e\right)\frac{dT_e}{dt} &= G_{e-ph}\left(T_l - T_e\right) + P_{exc}(t) \\ C_l\frac{dT_l}{dt} &= G_{e-ph}\left(T_e - T_l\right) \ , \end{aligned} \tag{S8}$$

where $T_{e,l}$ is the temperatures of the thermalised hot electron or phonon ensemble, $C_l = 2.54\ Jcm^{-3}K^{-1}$ is the heat capacity of of the crystal lattice taken here in the Dulong-Petit limit,[14] $C_e(T_e)$ is the temperature-dependent hot-carrier specific heat, and G_{e-ph} is the phenomenological electron-phonon coupling constant. Low carrier density and non-parabolicity of the band structure of ITO strongly affects the values and temperature dependence of C_e and G_{e-ph}.

The parameters of the TTM can be evaluated analytically using the Sommerfeld expansion up to 4th order of k_BT_e, following equations (25-26) from Ref.[15] The corresponding temperature dependences for ITO used in this work are shown in Fig. S5(a,b). While we used rigorously calculated G_{e-ph} to obtain the results in Fig. 3(a-c), we would like to note that this accuracy is likely excessive for the practical applications within the scope of the TTM, and the low-electron-temperature limit of this value can be used instead:[15]

$$G_{e-ph}\left(T_e \rightarrow 0K\right) = \frac{D^2 m_e^* k_B k_F^4}{\pi^3 \rho \hbar^3}\left(1 + 2CE_F\right)^2\ , \tag{S9}$$

where D = 17.2 eV is the deformation potential,[15] m_e^* is the effective electron mass in ITO, $\rho = 7.1\ gcm^{-3}$ is the ITO density. This limit is shown as a dashed orange line in Fig. S5(b). Figure S5(c) shows that the error in calculation imposed by omitting the temperature dependence of the electron-phonon coupling constant is not significant for ITO.

The source term appearing in Eq. (S8) is modelled as a Gaussian pulse:

$$P_{exc} = A(\lambda)\frac{F}{d\tau}\exp\left[-4\ln 2\left(\frac{t}{\tau}\right)^2\right]\ , \tag{S10}$$

where τ = 250 fs, d is the thickness of the ITO film, F is the pump fluence, and $A(\lambda)$ is the power absorption coefficient of ITO at the excitation wavelength. Since the pulses in this study are relatively long, they are not sensitive to non-equilibrium dynamics of the hot carriers in ITO, which would be important for shorter optical pulses.[15]

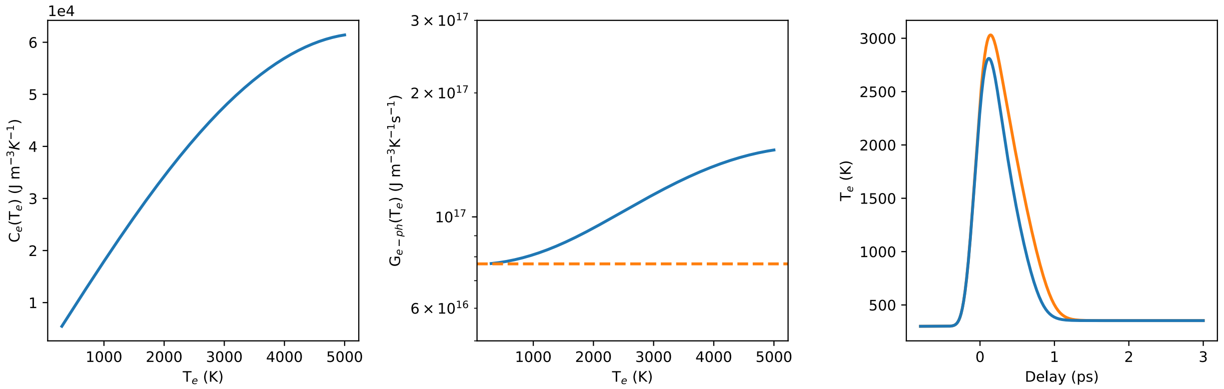

Figure S5: (a,b) Temperature dependence of ITO material parameters: (a) free-electron heat capacity and (b) electron-phonon coupling constant; dashed orange line in (b) shows the the low-electron-temperature limit (Eq. (S9)). (c) Temporal evolution of the hot-electron temperature calculated with the full expression for G_{e-ph} (blue) and in the low-electron-temperature limit (orange).

4.1 Electron-electron scattering and the optical properties of ITO

As described in the main text, the Drude model was used to treat the response of ITO under photoexcitation. While the main effect considered in this work is related to the shift of the plasma frequency (Eq. (1) from the main text), we also need to consider the contribution of the hot-electron-electron scattering to the damping constant in the Drude model, which is commonly disregarded for steady-state measurements but needs to be taken into account at elevated electron temperatures to describe the transient changes. For the non-parabolic conduction band of ITO, up to the second order of $k_B T_e$, the hot-electron energy dependence of the Drude damping constant is given by[15]

$$\gamma_{e-e}(\mathcal{E}) = \Lambda \left[(k_B T_e)^2 + \left(\frac{\mathcal{E}-\mu}{\pi}\right)^2 + \frac{C(\mathcal{E}-\mu)}{(1+2C\mu)^3} (k_B T_e)^2 + ... \right], \quad \text{(S11)}$$

$$\Lambda = \frac{m_e^* e^4}{64\pi\hbar^3 \epsilon_0^2, \epsilon_c^2 E_s^{3/2} E_\mu^{1/2}} \times \left[\frac{2\sqrt{E_s E_\mu}}{4E_\mu + E_s} + \arctan\sqrt{\frac{4E_\mu}{E_s}} \right] (1+2C\mu)^3 ,$$

where $\mu = \mu(T_e)$ is the temperature-dependent chemical potential, ϵ_c is the static permittivity related to the response of the core electrons, and E_s and E_μ are defined as

$$\begin{aligned} E_s &= \frac{\hbar^2 q_{TF}^2}{2m_e^*} = \frac{e^2\hbar^2}{2m_e^*\epsilon_0\epsilon_c} \int d\mathcal{E} \left(-\frac{\partial f}{\partial \mathcal{E}}\right) \rho_e\left(\mathcal{E}\right), \\ E_\mu &= \mu(T_e)\left(1 + C\mu(T_e)\right) \end{aligned} \tag{S12}$$

with q_{TF} being the Thomas-Fermi wave vector. The chemical potential of ITO can be found within the second-order of the Sommerfeld expansion as[15]

$$\begin{aligned} \mu(T_e) \approx E_F - \frac{(\pi k_B T_e)^2}{6\rho_e(E_F)} \frac{d\rho_e(\mathcal{E})}{\mathcal{E}}|_{\mathcal{E}=E_F} = \\ E_F - \frac{(\pi k_B T_e)^2}{12 E_F(1 + C E_F)} \frac{2(1 + 2CE_F)^2 - 1}{1 + 2CE_F}, \end{aligned} \tag{S13}$$

where C$=$ 0.42 eV^{-1} is the non-parabolicity factor and $\rho_e(\mathcal{E})$ is the electron density of states. The temperature dependence of the chemical potential of ITO is much stronger than in noble metals due to the lower carrier concentration.

Equations (S11–S12) closely resemble the well-known expressions for the hot-electron lifetimes in the ideal isotropic free-electron model.[16,17] However, the difference in effective mass, the $(1+2C\mu)^3$ term and the lower Fermi level results in a factor of 10 larger value of Λ, which is partially mitigated by a stronger screening of core electrons with $\epsilon_c \approx 9$.[1,2] Taking these factors into account, a value $\Lambda \approx 1.5$ eV^{-2}fs^{-1} is obtained, resulting in a stronger electron-electron scattering contribution to the Drude damping in ITO compared to Ag and Au for which $\Lambda \approx 0.3$ eV^{-2}fs^{-1}.

Finally, since elastic electron-electron collisions conserve a total current, only the wrap-around Umklapp processes contribute to the Drude damping coefficient. This "fractional Umklapp scattering" is usually introduced by multiplying Eq. (S11) by a factor $\Delta \approx 0.75$ in typical noble metals ($\Lambda \rightarrow \Gamma = \Delta \cdot \Lambda$).[18] Using this value of Δ for treating ITO, however, clearly overestimates the effect of damping observed in our experiment, and the lower value

of Δ is the key to explaining the inconsistency. Precise calculations of fractional Umklapp scattering are cumbersome and we are not aware of such calculations ever done for ITO. However, in the framework of Ref.,[19] fractional Umklapp scattering scales as m_e^*/m_e. Using this scaling, we obtain $\Delta \approx 0.3$ and $\Gamma = \Delta \cdot \Lambda \approx 0.48$ eV^{-2}fs^{-1}, which contributes to Eq. 2 in the main text. This value is also close to another estimate obtained from[20,21]

$$\gamma_{e-e} \approx \frac{\omega^2}{4\pi^2\omega_p}\left[1 + \left(\frac{2\pi k_B T_e}{\hbar\omega}\right)^2\right]. \tag{S14}$$

It should be noted that while Eq. (S11) includes a second term proportional to $(k_B T_e)^2$, the coefficient in front of it makes it approximately 10 times smaller than the leading term in the energy range $E_f \pm \hbar\omega$.

References

(1) Schleife, A.; Neumann, M. D.; Esser, N.; Galazka, Z.; Gottwald, A.; Nixdorf, J.; Goldhahn, R.; Feneberg, M. Optical properties of In_2O_3 from experiment and first-principles theory: influence of lattice screening. *New Journal of Physics* **2018**, *20*, 053016.

(2) Hamberg, I.; Granqvist, C. G. Evaporated Sn-doped In2O3 films: Basic optical properties and applications to energy-efficient windows. *Journal of Applied Physics* **1986**, *60*, R123–R160.

(3) Tamanai, A.; Dao, T. D.; Sendner, M.; Nagao, T.; Pucci, A. Mid-infrared optical and electrical properties of indium tin oxide films. *Physica Status Solidi (a)* **2017**, *214*, 1600467.

(4) Wells, B.; Bykov, A. Y.; Marino, G.; Nasir, M. E.; Zayats, A. V.; Podolskiy, V. A. Structural second-order nonlinearity in plasmonic metamaterials. *Optica* **2018**, *5*, 1502–1507.

(5) Sipe, J. E.; So, V. C. Y.; Fukui, M.; Stegeman, G. I. Analysis of second-harmonic generation at metal surfaces. *Phys. Rev. B* **1980**, *21*, 4389–4402.

(6) Rudnick, J.; Stern, E. A. Second-harmonic radiation from metal surfaces. *Phys. Rev. B* **1971**, *4*, 4274–4290.

(7) Corvi, M.; Schaich, W. L. Hydrodynamic-model calculation of second-harmonic generation at a metal surface. *Phys. Rev. B* **1986**, *33*, 3688–3695.

(8) Schaich, W. L.; Liebsch, A. Nonretarded hydrodynamic-model calculation of second-harmonic generation at a metal surface. *Phys. Rev. B* **1988**, *37*, 6187–6192.

(9) Liebsch, A. Second-harmonic generation at simple metal surfaces. *Phys. Rev. Lett.* **1988**, *61*, 1233–1236.

(10) Rodríguez-Suné, L.; Scalora, M.; Johnson, A. S.; Cojocaru, C.; Akozbek, N.; Coppens, Z. J.; Perez-Salinas, D.; Wall, S.; Trull, J. Study of second and third harmonic generation from an indium tin oxide nanolayer: Influence of nonlocal effects and hot electrons. *APL Photonics* **2020**, *5*, 010801.

(11) Shen, Y. *The Principles of Nonlinear Optics*; Wiley classics library; Wiley, 2003.

(12) Sipe, J. E. New Green-function formalism for surface optics. *JOSA B* **1987**, *4*, 481–489.

(13) Allen, P. B. Theory of thermal relaxation of electrons in metals. *Phys. Rev. Lett.* **1987**, *59*, 1460–1463.

(14) Un, I.-W.; Sarkar, S.; Sivan, Y. Electronic-based model of the optical nonlinearity of low-electron-density Drude materials. *Phys. Rev. Appl.* **2023**, *19*, 044043.

(15) Sarkar, S.; Un, I. W.; Sivan, Y. Electronic and thermal response of low-electron-density Drude materials to ultrafast optical illumination. *Phys. Rev. Appl.* **2023**, *19*, 014005.

(16) Del Fatti, N.; Voisin, C.; Achermann, M.; Tzortzakis, S.; Christofilos, D.; Vallée, F. Nonequilibrium electron dynamics in noble metals. *Phys. Rev. B* **2000**, *61*, 16956–16966.

(17) Ogawa, S.; Nagano, H.; Petek, H. Hot-electron dynamics at Cu(100), Cu(110), and Cu(111) surfaces: Comparison of experiment with Fermi-liquid theory. *Phys. Rev. B* **1997**, *55*, 10869–10877.

(18) Beach, R. T.; Christy, R. W. Electron-electron scattering in the intraband optical conductivity of Cu, Ag, and Au. *Phys. Rev. B* **1977**, *16*, 5277–5284.

(19) Lawrence, W. E.; Wilkins, J. W. Electron-electron scattering in the transport coefficients of simple metals. *Phys. Rev. B* **1973**, *7*, 2317–2332.

(20) Alam, M. Z.; Leon, I. D.; Boyd, R. W. Large optical nonlinearity of indium tin oxide in its epsilon-near-zero region. *Science* **2016**, *352*, 795–797.

(21) Minerbi, E.; Sideris, S.; Khurgin, J. B.; Ellenbogen, T. The role of epsilon near zero and hot electrons in enhanced dynamic THz emission from nonlinear metasurfaces. *Nano Letters* **2022**, *22*, 6194–6199.
